# Do N-Terminal Pro-C-Type Natriuretic Peptide Levels Relate to Severity of Preeclampsia?

**DOI:** 10.1155/2020/2693534

**Published:** 2020-05-02

**Authors:** Dogan Vatansever, Pınar Vatansever, Burak Giray, A. Aktug Ertekin, Serpil Bilsel

**Affiliations:** ^1^Koc University School of Medicine, Department of Obstetrics and Gynecology, Istanbul, Turkey; ^2^Marmara University Faculty of Medicine, Department of Biochemistry, Istanbul, Turkey; ^3^Zeynep Kamil Training and Research Hospital, Department of Obstetrics and Gynecology, Istanbul, Turkey; ^4^Uskudar University Faculty of Health Sciences, Department of Nursing, Istanbul, Turkey

## Abstract

**Aim:**

To compare the plasma N-terminal pro-C-type natriuretic peptide concentrations of normotensive pregnant women, patients with mild preeclampsia, and patients with severe preeclampsia.

**Methods:**

We collected venous blood samples from 25 normotensive pregnant women, 15 patients with mild preeclampsia, and 15 patients with severe preeclampsia. The women were at 30th to 40th weeks of gestation and in an age range of 20 to 35. The N-terminal pro-C-type natriuretic peptide levels were measured by ELISA. Statistical comparisons were made by one-way analysis of variance, Kruskal–Wallis, and Mann–Whitney *U* tests.

**Results:**

The median (interquartile range-IQR) values of the N-terminal pro-C-type natriuretic peptide were 6.48 (3.33) pmol/L in the normotensive women group, 7.37 (3.43) pmol/L in patients with mild preeclampsia, and 11.52 (6.13) pmol/L in patients with severe preeclampsia. The N-terminal pro-C-type natriuretic peptide was significantly elevated in the severe preeclampsia study group (*P* < 0.001), whereas there was no significant difference between those with mild preeclampsia and the normotensive groups (*P* > 0.05).

**Conclusion:**

Our data indicate that the plasma concentration of the N-terminal pro-C-type natriuretic peptide is significantly increased in patients with severe preeclampsia, but not in patients with mild preeclampsia. The severity of preeclampsia may be related to the circulating levels of the N-terminal pro-C-type natriuretic peptide concentrations.

## 1. Introduction

The natriuretic peptide family is composed of three principal peptides sharing the same 17 amino acid ring structure: A-type (atrial) natriuretic peptide, B-type (brain) natriuretic peptide, and C-type natriuretic peptide [1]. These natriuretic peptides form their biological effects via activation of three different natriuretic peptide receptors which have previously been described as natriuretic peptide receptors A, B, and C [2]. The A-type natriuretic peptide and the B-type natriuretic peptide bring about their biological effects through the natriuretic peptide receptor A, whereas the C-type natriuretic peptide acts more selectively with the natriuretic peptide receptor B. Both these natriuretic peptide receptors increase the intracellular levels of cyclic guanosine monophosphate, resulting in several different physiologic changes influencing a variety of homeostatic processes [3]. In contrast to natriuretic peptide receptors A and B, the natriuretic peptide receptor C acts as a clearance receptor. In addition to its clearance function, it also has important actions on blood vessel tonus [4].

The C-type natriuretic peptide was the third member of this family to be identified. The C-type natriuretic peptide is a locally acting peptide in an autocrine/paracrine fashion. It is present at very low concentrations in the plasma, as a consequence of its rapid clearance from the circulation [5]. It has a wide spectrum of biological actions such as vasodilation, growth inhibition in vascular smooth muscle cells [6], modulation of immune cell and platelet activities [7], and vascular remodeling. Also, elevated plasma C-type natriuretic peptide levels are found in patients with septic shock, heart failure, and renal dysfunction, thereby associating this endothelium-derived peptide with cardiovascular and endothelial pathologies [2].

Preeclampsia and eclampsia are two of the hypertensive disorders of pregnancy. The pathophysiological mechanisms in the development of these disorders are not clear yet although it is well known that endothelial dysfunction has a fundamental role [8]. As far as we are aware, there are two publications in the literature which compare the levels of the C-type natriuretic peptide of the patients with preeclampsia and normotensive healthy pregnant women. In one of these studies, C-type natriuretic peptide levels in the plasma were detected by the enzyme-linked immunosorbent assay (ELISA) and no significant difference could be found [9]. In the other study, plasma levels of the N-terminal pro-C-type natriuretic peptide (the longer half-life peptide precursor of the C-type natriuretic peptide) were evaluated and Prickett et al. found that plasma levels of the N-terminal pro-C-type natriuretic peptide were higher in patients with preeclampsia [10]. We aimed in this study to measure the plasma levels of the N-terminal pro-C-type natriuretic peptide in preeclamptic patients and analyze its relation to the severity of preeclampsia.

## 2. Materials and Methods

A total of 55 pregnant women followed in the Department of Obstetrics and Gynecology, Zeynep Kâmil Women and Children Diseases Education and Research Hospital, Turkey, were enrolled in this study. 15 patients with severe preeclampsia and 15 patients with mild preeclampsia were selected for the preeclamptic group. A written informed consent was obtained from all women. All women selected were between the ages of 20 and 35 and had singleton pregnancies. Those with diabetes, cardiac or renal disease, and chronic hypertension were excluded from the study. Also, women diagnosed with HELLP syndrome were excluded from the study. All women were also in their third trimester of gestation. None of the women was in labor. Arterial blood pressures were recorded for at least two different times 4 hours apart when the patient is on bed rest from the right arm using a standard sphygmomanometer with an appropriate cuff size by the same clinician. Korotkoff sound K5 (disappearance of all sounds) was used to determine diastolic blood pressure. Preeclampsia is defined by the minimum criteria of blood pressure ≥140/90 mm Hg after 20 weeks of gestation and proteinuria 300 mg/24 hours or 1+ dipstick, according to the report by the Report of the American College of Obstetricians and Gynecologists' Task Force on Hypertension in Pregnancy [11]. Twenty five healthy normotensive women composed the control group. The 25 women in the control group were selected in a matched fashion with the patients in the preeclamptic group according to age and gestational age. The thirty women who composed the preeclamptic group were further divided into severe and mild preeclamptic patient categories, each group formed by fifteen patients, according to the criteria defined in the same report such as

  Systolic blood pressure of 160 mm Hg or higher or diastolic blood pressure of 110 mm Hg or higher on two occasions at least 4 hours apart while the patient is on bed rest (unless antihypertensive therapy is initiated before this time)
  Thrombocytopenia (platelet count less than 100,000/microliter)
  Impaired liver function as indicated by abnormally elevated blood concentrations of liver enzymes (to twice normal concentration), severe persistent right upper quadrant or epigastric pain unresponsive to medication and not accounted for by alternative diagnoses, or both
  Progressive renal insufficiency (serum creatinine concentration greater than 1.1 mg/dL or a doubling of the serum creatinine concentration in the absence of other renal disease)
  Pulmonary edema
  New-onset cerebral or visual disturbances

One venous blood sample (10 mL) was collected from the patients with preeclampsia and the healthy controls into tubes containing ethylenediaminetetraacetic acid (K_2_EDTA). The blood samples of patients with preeclampsia were collected before they received any medication. Immediately after sampling, plasma samples were separated by centrifugation at 2000 × *g* for 15 min at 4°C and frozen at −80°C until assayed. The N-terminal pro-C-type natriuretic peptide levels in these plasma samples were measured with the ELISA method using the N-terminal pro-C-type natriuretic peptide EIA Kit (Biomedica, Austria). The lower detection limit was 0.55 pmol/L. The intraassay variations were 5-6% for a concentration range of 5.1–20 pmol/L, and the interassay variations were 1% for a concentration range of 5–20 pmol/L as stated by the manufacturer. All of the samples were run in a duplicate manner in the same assay. The laboratory staff were blinded to the clinical condition of the women in the study groups.

In this study, data were given as means ± standard deviation and medians (IQR). Repeated measures of one-way analysis of variance (ANOVA), Kruskal–Wallis, and Mann–Whitney *U* tests were used to assess the data, in which the significant difference between groups was defined as *P* < 0.05. Dunn's multiple comparisons test used as a *post hoc* test for the significant results. The power analysis has been done assuming that we have 14 patients in each group with a standard deviation of 4. Even in this scenario, the power calculated was 0.87. Since we have at least 15 patients in each study group, the power of this study seems sufficient.

Our study protocol was approved by the institutional review board.

## 3. Results

As shown in Table 1, there was no significant difference among the normotensive, mild preeclamptic, and severe preeclamptic study groups in terms of maternal age, gravida, parity, and body mass index (BMI). Also, the gestational ages did not differ among these groups. Patients with severe preeclampsia had significantly lower mean birthweights than normotensive patients and patients with mild preeclampsia (*P* < 0.05) (Table 1). The distinction of systolic and diastolic blood pressure measures was significant between all three groups, and the highest pressure being observed in the severe preeclamptic group.

**Figure 1:**
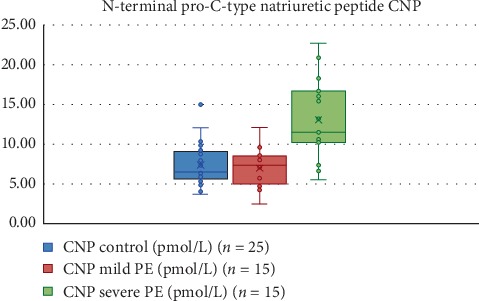
The spread of data among the patients with severe preeclampsia, the patients with mild preeclampsia, and the normotensive pregnant women.

**Figure 2:**
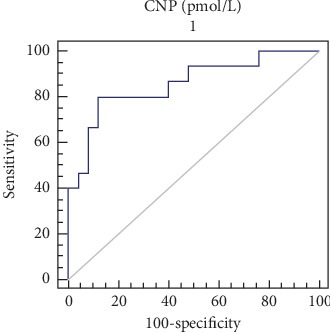
Receiver operator curve (ROC) analysis between the patients with severe preeclampsia and the normotensive pregnant women. AUC is 0.856 with a 95% confidence interval of 0.709 to 0.947 and a standard error of 0.0659 (*P* > 0.0001).

With regard to the biochemical findings presented in Table 2, there was an expected significant difference in proteinuria among all three groups. There was a significant difference between the normotensive group as compared with the other two study groups. Both the mild and severe preeclamptic patients had higher lactate dehydrogenase (LDH) levels than the normotensive women. There was no significant difference for aspartate transaminase (AST) and alanine transaminase (ALT) values among the three study groups. The plasma levels of the N-terminal pro-C-type natriuretic peptide were significantly elevated in the severe preeclampsia study group differing both from the normotensive (*P* < 0.001) and mild preeclampsia (*P* < 0.01) study groups, whereas there was no significant difference among the mild preeclampsia and normotensive study groups (*P* > 0.05). The median (IQR) values of the N-terminal pro-C-type natriuretic peptide were 6.48 (3.33) in the normotensive women group, 7.37 (3.43) in patients with mild preeclampsia, and 11.52 (6.13) in patients with severe preeclampsia. The spread of data among the study groups and the control group is displayed in Figure 1. Receiver operator curve (ROC) analysis showed that 10.17 pmol/L is the cutoff value for severe preeclampsia with an LR of 6.67, sensitivity of 80% (CI 51.9–95.7), and specificity of 88% (CI 68.8–97.5) (Figure 2).

## 4. Discussion

The C-type natriuretic peptide was the third member of the natriuretic peptide family to be discovered. The natriuretic peptide family has important effects on the control of blood pressure, volume, and the management of the renal function [9]. The C-type natriuretic peptide, the most widely expressed natriuretic peptide, seems to be one of the most important members of this family in the regulation of blood pressure. It has been shown that a polymorphism in the three prime untranslated region (3′-UTR) of the C-type natriuretic peptide gene (*Nppc*) is associated with hypertension in a Japanese study [12]. Allelic variants in the three prime untranslated region of natriuretic peptide receptors A and C have also been found to be associated with familial hypertension and higher systolic blood pressure [13]. Furthermore, administration of the exogenous C-type natriuretic peptide causes a dramatic drop in blood pressure in humans as well as some other species such as primates and dogs [2]. The C-type natriuretic peptide also plays a crucial role in chronic regulation of blood pressure by suppression of sympathetic outflow [14] and adrenocorticotropic hormone [15], aldosterone [15], and vasopressin synthesis [16]. Preeclampsia is a syndrome affecting nearly every organ system and is characterized by hypertension and proteinuria; however, the changes in C-type natriuretic peptide levels in relation to preeclampsia are not yet clear. As far as we are aware, there are two publications in the literature comparing the levels of the C-type natriuretic peptide between preeclamptic patients and normotensive healthy pregnant women. The levels of the C-type natriuretic peptide in preeclamptic patients were first investigated by Stepan et al. In this study, the plasma concentration of the C-type natriuretic peptide was measured in preeclamptic patients, pregnant women with gestational hypertension, and healthy pregnant subjects. They were unable to show significant differences between these groups [9]. The second study conducted by Prickett et al. investigated the plasma concentrations of the N-terminal pro-C-type natriuretic peptide in preeclamptic and normotensive pregnant women, at the time of delivery. Since the C-type natriuretic peptide is a locally acting peptide with a very short half-life in circulation, they used the longer half-life peptide precursor of the C-type natriuretic peptide, which is the N-terminal pro-C-type natriuretic peptide. In Prickett's study, 23 subjects, 13 of which diagnosed as preeclamptic without discrimination according to the severity of the syndrome, were evaluated, and they found significant differences among the two groups [10].

In our study, we compared the plasma N-terminal pro-C-type natriuretic peptide concentrations of 55 subjects, composed of 25 normotensive healthy pregnant women and 30 pregnant women with preeclampsia, all of which were in their 30–40 weeks of gestational ages. The preeclamptic study group was further divided into severe and mild preeclamptic groups, each composed of 15 patients, discriminated according to the criteria defined in the report by the Report of the American College of Obstetricians and Gynecologists' Task Force on Hypertension in Pregnancy [11]. We found that the plasma concentrations of the N-terminal pro-C-type natriuretic peptide were significantly higher in the severe preeclamptic study group in contrast to both mild preeclamptic and normotensive study groups, but there was no significant difference between the latter groups. Also, the mean plasma concentrations of the N-terminal pro-C-type natriuretic peptide in all three groups were lower than the concentrations previously noted by Prickett et al. This could be due to the timing of blood sampling which was at the time of delivery in this study. It has been previously shown that in labor the C-type natriuretic peptide was significantly upregulated [17]. Also, in a study by Prickett et al., it has been showed that the mean N-terminal pro-C-type natriuretic peptide levels were significantly lower in pregnant women compared to nonpregnant women, and these levels fall throughout the pregnancy and remain below than those found in age-matched nonpregnant women until at least 36 weeks. These values are more consistent with our data [18].

Our data indicate that the severity of preeclampsia could be related to the circulating levels of the N-terminal pro-C-type natriuretic peptide. Espiner et al. indicated that preeclampsia and GHT with SGA were associated with raised maternal plasma NTproCNP [19]. Since the endothelial dysfunction is one of the most important mechanisms both in the pathophysiology of preeclampsia and the endothelial release of the N-terminal pro-C-type natriuretic peptide, this end point seems logical. Even though these findings are noteworthy, it is unclear if the increased levels of the N-terminal pro-C-type natriuretic peptide could be useful in the prediction or detection of severe preeclampsia in clinical use. One reason for this is that the increase in plasma levels of the N-terminal pro-C-type natriuretic peptide seems likely to be the result of endothelial dysfunction which also aggravates preeclampsia. A prospectively designed study might provide the answer to whether we can predict the more severe cases of preeclampsia which will most probably need further treatment and hospitalization by following N-terminal pro-C-type natriuretic peptide levels. Notably, there are numerous emerging studies on the prognostic and therapeutic role of ANP and BNP, and the other two members of the natriuretic peptide family in cardiovascular diseases. Clinical trials have noted the benefits and risks of the synthetic A-type natriuretic peptide (anaritide) and the B-type natriuretic peptide (nesiritide) usage for treating hypertension, heart failure, and renal failure [20].

## 5. Conclusion

Since we know that C-type natriuretic peptide infusion drops blood pressure, it may also find a therapeutic use in treatment of hypertension in pregnant women. It is evident that further studies are needed to clarify the prognostic and therapeutic role of the C-type natriuretic peptide in preeclamptic patient population.

## Figures and Tables

**Table 1:** Patient characteristics.

Variable	Normotensive patients (*n* = 25)	Patients with mild preeclampsia (*n* = 15)	Patients with severe preeclampsia (*n* = 15)
Maternal age (year)	27.9 ± 4.8	29.6 ± 3.8	26.7 ± 5.2
Gravida	3 (1–6)	2 (1–8)	3 (1–4)
Parity	2 (0–4)	2 (0–6)	2 (1–3)
Body mass index of mother (kg/m^2^)	28.33 ± 3.8	26.21 ± 3.2	28.64 ± 2.8
Birthweight (gram)	3371 ± 484	3106 ± 538	2656 ± 614^*∗*^
Gestational age (week)	35.6 ± 1.9	35.5 ± 2.7	35.6 ± 1.7
Systolic blood pressure (mmHg)	100 (10)	140^*∗∗*^ (10)	160^*∗∗*^ (10)
Diastolic blood pressure (mmHg)	70 (20)	90^*∗∗*^ (2.5)	100^*∗∗*^ (7.5)

The values are presented as mean ± standard deviation and medians (IQR values). ^*∗*^*P* < 0.05 versus normotensive women and patients with mild preeclampsia. ^*∗∗*^*P* < 0.05 versus normotensive women.

**Table 2:** Biochemical findings and plasma N-terminal pro-C-type natriuretic peptide concentrations.

Variable	Normotensive patients (*n* = 25)	Patients with mild preeclampsia (*n* = 15)	Patients with severe preeclampsia (*n* = 15)
Proteinuria (mg/24 h)	—	613.8 ± 306.5	3842.5 ± 2122.9†
Plasma creatinine (mg/dL)	0.5 (0.1)	0.6^*∗*^ (0.2)	0.7^*∗*^ (0.1)
LDH (U/L)	151.6 (37)	219.6^*∗*^ (79.7)	277.6^*∗*^ (66.9)
AST (U/L)	17.2 (11.8)	21.2 (8.4)	21.4 (12.8)
ALT (U/L)	11.6 (4.6)	12.3 (8.0)	12.9 (7.8)
N-terminal pro-C-type natriuretic peptide (pmol/L)	6.48 (3.33)	7.37 (3.43)	11.52^*∗*^, ^*∗∗*^(6.13)

The values are presented as mean ± standard deviation and medians (IQR values). ^*∗*^*P* < 0.05 versus normotensive women. ^*∗∗*^*P* < 0.05 versus mild preeclamptic women. ^†^*P* < 0.05 versus both normotensive and mild preeclamptic women.

## Data Availability

All data used to support the findings of this study are available from the corresponding author upon request.
